# Evaluation of the Psychosocial Status of Patients With Type 2 Diabetes Mellitus and Their Treating Physicians Using the Glycemic Happiness Scale: A Multicentric, Cross-sectional Study

**DOI:** 10.7759/cureus.80209

**Published:** 2025-03-07

**Authors:** Sanjay Kalra, Abhay K Sahoo, Shehla Shaikh, Vaishali Deshmukh, Shreerang Godbole, Ameya Joshi, Jayashree Swain, Prasanna Kumar K M, Vijaya B Reddy Sagili, Radha Rani P, Rajnish Dhediya, Rajan Mittal, Bhavesh P Kotak, Gauri Dhanaki

**Affiliations:** 1 Endocrinology, Bharti Research Institute of Diabetes & Endocrinology (BRIDE), Karnal, IND; 2 Endocrinology, Institute of Medical Sciences and Sum Hospital, Bhubaneswar, IND; 3 Endocrinology, KGN Diabetes and Endo Centre, Mumbai, IND; 4 Endocrinology, Saifee Hospital, Mumbai, IND; 5 Endocrinology, Deenanath Mangeshkar Hospital and Research Centre, Pune, IND; 6 Endocrinology and Diabetes, INSTRIDE, Pune, IND; 7 Endocrinology, Bhaktivedanta Hospital, Mumbai, IND; 8 Department of Endocrinology, Diabetic and Endocare Clinic, Bhubaneswar, IND; 9 Endocrinology and Diabetes, Bangalore Diabetes Centre, Bengaluru, IND; 10 Endocrinology, Diabetes and Metabolism, Vijay Diabetes, Thyroid, and Endocrine Clinic, Puducherry, IND; 11 Endocrinology, Sri Sai Ram Endocrine and Diabetic Center, Kurnool, IND; 12 Department of Medical Affairs, Dr. Reddy’s Laboratories Ltd., Hyderabad, IND; 13 Department of Medical Affairs, Dr. Reddy's Laboratories Ltd., Hyderabad, IND; 14 Clinical Research, Dr. Reddy's Laboratories Ltd., Hyderabad, IND

**Keywords:** gh scale, glycemic happiness, oral antidiabetic drugs (oad), psychosocial status, type 2 diabetes

## Abstract

Background: The purpose of this study was to evaluate the psychosocial status of patients with type 2 diabetes (T2D) and treating physicians using a novel Glycemic Happiness (GH) scale.

Methods: This was a real-world, prospective, multicentric, cross-sectional observational study. Male and female participants aged ≥ 18 years with a clinical diagnosis of T2D were eligible to take part. Additionally, the physicians with extensive T2D patient experience were enrolled in the study.

Results: The study included a total of 400 participants of T2D and 27 physicians. The patient population consisted of 213 (53.3%) men and 187 (46.7%) women, with a mean age of 54.29 ± 12.05 years. The proportions of patients and physicians who were found to be happy were 50.5% and 90.8%, respectively. A statistically significant difference was found in the mean value of the GH score of insulin and oral antidiabetic drugs (OAD) for the physician component (P=0.0160). Although no significant difference in GH score was observed between insulin and OAD usage among the patients (P=0.9564), a significant difference was observed for dosage frequency of OAD (Once daily (OD) vs. three times daily (TID) (P=0.0034) and twice daily (BID) vs. TID (P=0.0324)). In the multiple regression analysis, GH in patients was found to be associated with glycated hemoglobin (HbA1c) and postprandial glucose (PPG) values.

Conclusions: High glucose levels and OAD dosage frequency have been negatively associated with GH in patients with T2D. To achieve long-term happiness and well-being in diabetes management, there is a need to focus on various aspects of GH among patients and physicians.

## Introduction

The global prevalence of type 2 diabetes (T2D) is on the rise, and it is expected to grow from 6059 cases per 100,000 population in 2017 to 7079 cases per 100,000 population by 2030 [1]. The ICMR-INDIAB nationwide cross-sectional population-based study of 113043 adults aged 20 years and older found an overall prevalence of diabetes and prediabetes in India at 11.4% and 15.3%, respectively [2]. It is estimated that by 2045, India will have 124.9 million adults with diabetes, accounting for 16% of the global disease burden [3]. Diabetes is commonly believed to be associated with social, emotional, behavioral, and environmental aspects, all of which impact psychological “well-being” and treatment success [4]. As a result, the World Health Organization (WHO) also emphasizes the need to consider emotional and mental health in diabetes patients in addition to physical health [5].

In a study to benchmark diabetes-related psychosocial effects across nations, 38.5% of Indian subjects experienced diabetic distress, placing India 11th out of 17 countries [6]. Happiness is an essential component of human health and a comprehensive psychological indicator for quantifying the QoL [7]. Glycemic Happiness (GH) is characterized as “an emotional and biomedical state of well-being in individuals with T2D” [5]. The Diabetes Distress Scale (DDS) and the Problem Areas in Diabetes (PAID) are two scales that are widely accepted as statistically sound measures of diabetes distress but they have certain limitations [8-9]. Several other tools, including the Hypoglycemia Attitudes and Behavior Scale, the Hypoglycemic Confidence Scale, the Diabetes Treatment Satisfaction Questionnaire, and the Self-Efficacy for Diabetes Scale, exist for assessing different aspects of happiness or well-being in both nondiabetic and diabetic settings [5], but none of them measure GH. While DDS and PAID mainly focus on diabetes distress and emotional distress, the novel GH scale additionally includes social and psychological components. Most people living with diabetes are unhappy with the care and services provided by their healthcare providers (HCPs), which might undermine their commitment to optimal self-care behavior [5,6]. As a result, it can be asserted that deliberate efforts by HCPs to promote GH in people living with diabetes will increase patient satisfaction, which is critical for improving diabetes outcomes [5,10]. Therefore, the purpose of this study was to evaluate the psychosocial status of patients with T2D and treating physicians using a novel GH scale.

## Materials and methods

Study design and eligibility criteria

This was a real-world, prospective, multicentric, cross-sectional observational study. The psychosocial status of patients and treating physicians was evaluated independently using the respective components of the GH scale. The participating investigators were required to be endocrinologists, diabetologists, or consulting physicians who treated a high number of T2D patients and demonstrated an interest in participating in the study. At each participating site, male and female patients aged 18 years or older with a clinical diagnosis of T2D as per the American Diabetes Association (ADA) guidelines were eligible for participation [11]. This study had enrolled the participants across the regions of India to ensure geographic diversity. Participants were excluded if they were unable or unwilling to provide informed consent and comply with the study procedures. The study was conducted in compliance with the ethical principles outlined in the most recent edition of the Declaration of Helsinki and the applicable guidelines for good clinical practice. The study was granted approval (Approval number: RPIEC270722) by the ethics committee at every participating site and registered on the Clinical Trials Registry of India portal with registration number CTRI/2022/09/045216.

Study Procedures - The GH scale

The GH scale used in this study as a tool to measure happiness in diabetes care was formulated to adapt to the existing practices and beliefs of Indian T2D patients and physicians. An expert group of nine Indian diabetologists and endocrinologists gathered and researched 17 globally validated scales [5] to reach a consensus on a set of questions (10 questions each for patients and physicians) that closely match the social and psychological components necessary in the GH scale. These scales consisted of questionnaires assessing treatment satisfaction, quality of life goals, and caregiver burden [5]. These GH scales were all patient-administered questionnaires to identify the key factors in diabetes care to define the overall GH of a person with T2D [6]. This formulated scale included two sets of questionnaires, one for the patient and the other for the physician (Appendices: Tables 7-8). The GH scale for the patient component had 10 questions, i.e., how satisfied are you with your understanding of your diabetes; do you feel friends or family don’t appreciate how difficult living with diabetes can be; how happy and satisfied are you with your life right now; how flexible have your treatment been lately; how convenient have your treatment been lately; how confident do you feel that you know what to do when your blood sugar level goes higher or lower than it should be; do you feel that diabetes has affected your private and social leisure activities; do you feel that diabetes is taking up too much of your mental and physical energy every day; do you get angry, scared, and/or depressed when you think about living with diabetes; and do you feel overwhelmed by the demands of living with diabetes. Likewise, the GH scale for the physician component also had 10 questions, i.e., do you feel happy and satisfied that you chose to be a diabetes care professional; do you get satisfaction from being able to help persons with T2D; do you feel you can make a difference in the life of persons with T2D through your work; do you get physically and emotionally exhausted at work; do you feel you are losing enthusiasm at work; do you feel you are in control of dealing with complex problems of T2D management; do you feel worn out by your job as a care provider; do you feel overwhelmed because persons with diabetes’ loads seem endless; do you feel depressed by the traumatic stress of persons with T2D that you try to help; and do you feel less empathetic and connected with your colleagues and friends. A five-point Likert scale was used to rate each question, with 1 representing “strongly disagree”, 2 representing “disagree”, 3 representing “neutral response (neither agree nor disagree)”, 4 representing “agree”, and 5 representing “strongly agree”. The score for each question was summed up to derive the total score out of 50 for either of the components. A score of 40 to 50 reflects a happy person, 30 to <40 represents a neutral person, and 0 to <30 represents an unhappy person. Both the patient and the physician filled out a GH scale for their respective components i.e., the patient component and the physician component.

Clinical data collected from participating patients included demographics, diabetes duration, glycemic parameters, treatment details, comorbid conditions, and other risk factors. Data collected from the participating physicians included their years of experience in managing T2D patients. The GH scale questionnaire was administered in person at the study site to both patients and physicians. Data were recorded in the electronic case report form (e-CRF) and securely submitted via the Internet through encrypted websites. The study adhered to designated standard operating procedures and was closely monitored throughout. During the Site Initiation Visit, the monitor provided training to the investigators and site personnel on the use of the e-CRF. Additionally, each center was supplied with a manual detailing the data entry procedure. The data collection, data analysis, and manuscript preparation were done by the clinical research organization (CRO) independently, while all the authors were involved in the review of the clinical study report and manuscript.

The primary study objective was to assess the psychosocial status of T2D patients and their treating physicians using this formulated scale. The secondary objectives were to investigate the association between baseline demographic and disease characteristics (age, gender, duration of diabetes, risk factors, HbA1c level, and medication frequency) and GH, compare GH between injectable and oral treatments, and assess GH with the frequency of administration of OADs and insulin.

Statistical analysis

Continuous variables were analyzed using arithmetic mean, standard deviation (SD), minimum maximum, and median. Frequencies and percentages were used to summarize categorical and nominal data. For both the patient and physician components, a subgroup analysis was carried out to evaluate the differences in GH between the insulin and OADs groups, as well as the relationship between GH and the frequency of administration (Once daily (OD), twice daily (BID), or three times daily (TID)) of OADs and insulin.

Multiple regression analysis was used to identify the association factors to explore variable correlation with scores on the GH scale. Variables with P<0.20 in bivariate analyses have been included in multivariate modeling. A univariate regression analysis was conducted to examine the relationship between GH satisfaction scores and various factors, including age, gender, alcohol consumption, comorbid conditions, duration of diabetes, fasting plasma glucose, HbA1c levels, obesity, postprandial glucose levels, resident status, and tobacco consumption. The backward stepwise elimination method has been used for model development in multiple regression analysis. Criteria for entry and removal of variables have been based on the likelihood ratio test, with entering and removing limits set at P<0.05 and P>0.10. The association variables have been identified in terms of coefficients beta with associated P-value. A two-sided P-value of less than 0.05 has been considered statistically significant. The statistical analysis was performed using XLSTAT version 2021.3.1 (ADDINSOFT SAS, Paris, France)/R version 4.0.5 statistical software.

## Results

Descriptive characteristics of the study population

The study included a total of 400 participants with a clinical diagnosis of T2D. The clinical data were collected from March 2022 to September 2023 (18 months) across 27 study sites. Each study site collected data from around 12 to 20 participants. Among the participating patients, there were 213 (53.3%) males and 187 (46.7%) females with a mean age of 54.29 ± 12.05 years. Of the 400 patients, 363 (90.7%) were on OADs, 33 (8.3%) were on insulin + OADs, and 4 (1%) were only taking insulin. The descriptive characteristics of the study population are presented in Table 1.

**Table 1:** Descriptive characteristics of the study population ^¥^Only 236 of the 400 patients had a record of all three HbA1c, FPG, and PPG values. SD: standard deviation; HbA1c: glycated hemoglobin; FPG: fasting plasma glucose; PPG: post-prandial plasma glucose; OADs: oral antidiabetic drugs; OD: once a day; BID: twice a day; TID: thrice a day

Variable	All (N=400)
Age, mean ± SD, year	54.29 ± 12.05
Sex, n (%)	
Male	213 (53.3)
Female	187 (46.7)
Comorbidities, n (%)	
Hypertension	161 (40.3)
Dyslipidemia	50 (12.5)
Hypothyroidism	50 (12.5)
Cardiovascular Disease	42(10.5)
Chronic Kidney Disease	8 (2.0)
Other	35 (8.7)
Duration of Diabetes, n (%)	
<1 year	44 (11.0)
1-5 years	111 (27.8)
6-10 years	100 (25.0)
>10 years	145 (36.3)
Smoking History, n (%)	
Yes	75 (18.7)
No	325 (81.3)
Alcohol Consumption, n (%)	
Yes	72 (18.0)
No	328 (82.0)
Obesity, n (%)	
Yes	126 (31.5)
No	274 (68.5)
Glycemic Parameters^¥^, mean + SD	
HbA1c	8.03 ± 1.513
FPG	144.17 ± 49.369
PPG	206.08 ± 74.997
Antidiabetic Medication, n (%)	
Insulin	04 (1.0)
Insulin + OADs	33 (8.3)
OADs	363 (90.7)
Medication Frequency, n (%)	
OD	195 (48.7)
BID	188 (47.0)
TID	16 (4.0)
Once a week	01 (0.3)

Additionally, the participating physicians from all 27 participating sites provided written consent for participation and completed the GH scale for the physician component. The participating physicians had an average of 12.96 ± 5.68 years of experience.

Summary of GH score

Table 2 summarizes the GH score for the patient component.

**Table 2:** Summary of GH Score for Patient and Physician Components (N=400) ^¥^A score of 40 to 50 reflects a happy person, 30 to <40 represents a neutral person, and 0 to <30 represents an unhappy person. SD: standard deviation; GH: glycemic happiness

Description	Total Score for Patient Component	Total Score for Physician Component
Mean ± SD^¥^	38.66 ± 7.038	45.90 ± 4.842
Happy, n (%)	202 (50.5%)	363 (90.8%)
Neutral, n (%)	161 (40.2%)	31 (7.8%)
Unhappy, n (%)	37 (9.3%)	6 (1.5%)

Of all patients, 202 (50.5%) reported being happy, 37 (9.3%) reported being unhappy, and 161 (40.3%) reported being neutral. Of the 400 patients, 363 (90.75%) were on only OADs, whereas 37 (9.25%) were on insulin, either with or without OADs. Within the insulin subgroup, OD, BID, and TID dosages were administered to 15 (40.54%), 20 (54.05%), and 2 (5.4%) of the patients, respectively. In the OADs subgroup, the patients with OD, BID, and TID dosage frequencies were 180 (49.58%), 168 (46.28%), and 14 (3.85%), respectively. Table 3 compares the patient component's GH score between the insulin and OAD groups.

**Table 3:** Comparison of GH Score Between Insulin and OADs Group *Significant ^¥^One subject was on once-a-week dosage of Dulaglutide GH: glycemic happiness; OADs: oral antidiabetic drugs; SD: standard deviation; OD: once a day; BID: twice a day; TID: thrice a day

Description	Insulin		OADs		P-value
	N	Mean + SD	N	Mean + SD	
Patient Component					
Overall Population	37	38.59±7.30	363^¥^	38.66±7.02	0.9564
OD Dose	15	39.40±8.25	180	37.95±6.98	0.4471
BID Dose	20	38.25±6.75	168	39.17±7.17	0.5849
TID Dose	2	36.00±8.49	14	42.00±4.11	0.1042
Physician Component					
Overall Population	37	47.41±3.74	363^¥^	45.74±4.92	0.0160*
OD Dose	15	46.67±3.72	180	44.94±5.00	0.1930
BID Dose	20	47.80±3.91	168	46.43±4.70	0.2134
TID Dose	2	49.00±1.41	14	47.50±5.05	0.6905

There was no statistically significant difference in the mean value of the GH score of insulin and OAD for the patient component in the overall population (P=0.9564). Similarly, there was no statistically significant difference in the mean value of the GH score for the insulin subgroup vis-a-vis frequency of OD, BID, or TID dosing (Table 4).

**Table 4:** Comparison of GH Score vis-a-vis Frequency of Dosing in Insulin and OADs Subgroup *Significant; ^¥^One subject was on once-a-week dosage of Dulaglutide GH: glycemic happiness; OADs: oral antidiabetic drugs; SD: standard deviation; OD: once a day; BID: twice a day; TID: thrice a day

Description	Insulin		OADs	
	N	Mean + SD	N	Mean + SD
Patient Component				
OD Dose	15	39.40±8.25	180	37.95±6.98
BID Dose	20	38.25±6.75	168	39.17±7.17
TID Dose	2	36.00±8.49	14	42.00±4.11
P-value Between				
OD vs. BID		0.6632		0.1084
BID vs. TID		0.7723		0.0324*
OD vs. TID		0.6719		0.0034*
Physician Component				
OD Dose	15	46.67±3.72	180	44.94±5.00
BID Dose	20	47.80±3.91	168	46.43±4.70
TID Dose	2	49.00±1.41	14	47.50±5.05
P-value Between				
OD vs. BID		0.3896		0.0043*
BID vs. TID		0.4326		0.4577
OD vs. TID		0.1781		0.0873

The difference in GH score for the OAD subgroup between OD and TID (P=0.0034) and between BID and TID (P=0.0324) was, nevertheless, statistically significant (Table 4).

The GH score for the physician component is summarized in Table 2. The proportion of physicians who were happy, unhappy, or neutral was 363 (90.8%), 6 (1.5%), and 31 (7.8%), respectively. Table 3 compares the physician component's GH score between the insulin and OAD groups. For the physician component, there was a statistically significant difference in the mean value of the GH score of insulin and OAD in the overall population (P=0.0160). There was no statistically significant difference in the mean value of GH score for the insulin subgroup vis-a-vis frequency of OD, BID, or TID dosing (Table 4). However, the difference in GH score for the OAD subgroup between OD and BID (P=0.0043) was found to be statistically significant (Table 4).

Association of the individual factors and achievement of GH

A univariate regression analysis was performed to check the association between the baseline demographic and disease characteristics (age, gender, alcohol consumption, comorbidities, duration of diabetes, fasting plasma glucose (FPG), HbA1c level, obesity, PPG, and tobacco consumption) and GH for the patient component (Table 5).

**Table 5:** Univariate association between total GH score for patients and patient characteristics *Significant GH: glycemic happiness; FPG: fasting plasma glucose; HbA1c: glycated hemoglobin; PPG: postprandial glucose

Parameters	Unstandardized Coefficient ß	Standard Error	Standardized Coefficient ß	P-value
Age	0.072	0.029	0.123	0.0137*
Alcohol (Yes/No)	-2.747	0.907		0.0026*
Comorbid conditions	0.816	0.720		0.2580
Duration of diabetes	0.002	0.004	0.024	0.6377
FPG	-0.031	0.008	-0.215	0.0001*
Gender (Male/Female)	-0.638	0.705		0.3664
HbA1c	-1.167	0.251	-0.251	
Obesity (Yes/No)	-1.049	0.757		0.1665
PPG	-0.031	0.005	-0.331	
Resident status (Urban/Rural)	1.702	0.894		0.0577
Tobacco (Yes/No)	-2.332	0.895		0.0095*

The regression analysis showed that age, alcohol consumption, FPG, HbA1c, PPG, and tobacco consumption have a significant association (P<0.05) with GH for the patient component. This positive unstandardized beta coefficient suggests that as participants' age increases, their reported satisfaction tends to increase as well. The negative beta coefficient suggests that participants’ variables like alcohol consumption, FPG, HbA1c, PPG, and tobacco consumption have an inverse association with the GH score. Using the backward variables selection method, five variables were retained in the model and HbA1c, and PPG variables were found to be statistically significant (Table 6).

**Table 6:** Multiple (stepwise) regression analysis for total GH score for patients and patient characteristics *Significant GH: glycemic happiness; HbA1c: glycated hemoglobin; PPG: postprandial glucose

Source	Value	Standard error	t	Pr>|t|	Lower bound (95%)	Upper bound (95%)
Age	0.08	0.05	1.57	0.12	-0.02	0.17
HbA1c	-0.12	0.05	-2.33	0.02*	-0.22	-0.02
PPG	-0.22	0.05	-4.20	<0.0001*	-0.32	-0.12
Alcohol-No	0.17	0.09	1.89	0.06	-0.01	0.35
Alcohol-Yes	0.09	0.09	0.96	0.34	-0.09	0.26

## Discussion

The impact of diabetes-related psychosocial deficiencies on longevity and health-related quality of life (HRQOL) is significant. About one-third of people living with diabetes report mental health problems and a lack of happiness at some point in their lives [12]. This is because a diagnosis of diabetes imposes additional self-monitoring requirements, as a result of which an individual's daily life undergoes considerable changes, resulting in added stress [13]. Despite the chronic nature of diabetes, the notion of GH gives people living with diabetes hope that they can live productive and enjoyable lives without fear of disease [5,6]. As a result, we conducted this study using a novel GH scale to assess the psychosocial status of T2D patients and their treating physicians.

In our study, we found a greater number of male patients, with a mean age of 54.29±12.05 years, which was in line with the findings of our previous study [6]. Furthermore, a higher percentage of participants (81.3% and 82%, respectively) were nonsmokers and non-alcoholics, which is comparable with the findings of another study (80.3% and 87.8%) that investigated diabetes distress, happiness, and associated factors in T2D patients [7].

In our study, the GH score for the patient component indicated that 50.5% of patients were happy, while 40.2% and 9.3% were either neutral or unhappy. The global survey incidence of the American Diabetes Association [14] and the DAWN2 study [15], both revealed psychological distress related to diabetes in 18%-45% and 46% of patients, respectively. A high prevalence of diabetes distress, depression, and stress was found in several other studies, with around 35% of people living with diabetes having high stress levels and 38-56.8% of the people living with diabetes having depression [10, 16-18]. Furthermore, previous studies showed that most people living with diabetes had a "slightly negative" to “very much negative” impact on their emotional well-being (47.5%) and connection with near and dear ones (29.1%) [19]. A study by Liu et al. found that when using the WHO-5 well-being index, 34.85% of diabetes patients experienced a decrease in happiness [7]. Although the number of patients in the neutral or unhappy category in our study is comparable to previous findings, the proportion of patients in the unhappy category alone is substantially lower. This might be due to the inherent differences in the methods employed to quantify happiness. Furthermore, the mean GH score of our study indicated a neutral (38.66 ± 7.038), rather than a happy response, which is consistent with the findings of our previous pilot study [6]. Moreover, in a pilot study, a moderate level of satisfaction was recorded in 54.4% of people living with diabetes [20] and our results are consistent with this finding.

It has been proven that in the multidisciplinary care of diabetes, factors associated with both patients and HCPs may affect and contribute to GH in either a favorable or negative way [6]. Physicians' happiness and job satisfaction have a significant impact on their patients, and burnout is linked to patient dissatisfaction [21]. The concept of “GH” is to prevent compassion fatigue and burnout in patients/HCPs [6]. Compassion fatigue is a comprehensive term that encompasses burnout and secondary traumatic stress (STS) [5,22-23]. Studies have demonstrated that stress, fatigue, and burnout among physicians have a negative influence on the quality of patient care [5,22-23]. The literature describes a variety of scales for measuring compassion fatigue, burnout, and quality of communication among physicians [5]. According to the self-assessment compassion fatigue scale, compassion fatigue can be assessed using a 9-item questionnaire that may be responded to with “yes” or “no”. If the physician answers “yes” to ≥4 questions, it may suggest compassion fatigue [5]. The ideal remedy for compassion fatigue would be to minimize burnout and STS while increasing compassion satisfaction, which is a positive characteristic of professional quality of life [23]. The mean GH score of the physician component in our study was 45.90 ± 4.842, with an overall response rate of 90.8% as happy, 7.8% as neutral, and 1.5% as unhappy. A high mean score of the physicians in a previous study suggested a positive association with GH for happiness and satisfaction as a diabetes care professional (4.9 ± 0.4), conviction in being able to make a difference in the lives of T2D patients (4.8 ± 0.4), and confidence in managing complex T2D problems (4.5 ± 0.6) [6]. Furthermore, T2D patients’ GH may be influenced by their physician’s confidence in T2D treatment, professional delight, and capacity to cope with physical and emotional stress, in addition to effective glucose control [24]. In our study, the physician component’s high mean GH score was associated with low burnout and compassion fatigue.

GH is positively associated with higher mean scores of treatment flexibility and convenience (4.2±0.8 and 4.2±0.7, respectively), as reported by Kalra et al. [6]. Furthermore, a significant difference (P=0.0160) in the mean value of the GH score between the insulin and OADs group was identified for the physician component. A significant difference in GH score between OD and BID dose (P=0.0043) was observed in the OADs group. We did not find a significant difference in the mean value of the GH score of insulin vs. OADs for the patient component (38.59±7.30 vs. 38.66±7.02; P=0.9564), which is consistent with previous reports in which no significant (P>0.05) difference in happiness was observed between insulin and OAD treatments [7]. For OD, BID, and TID dosage, there was a decreasing trend in the mean value of GH score in the insulin group and an increasing trend in the OADs group, although this did not reach a statistically significant level. Furthermore, only OADs showed a significant change in GH score when OD and BID dosage frequencies were compared to TID. The preference of patients for the oral route of administration and less frequent dosage to maintain compliance and comfort may lead to this type of trend. According to studies, the use of insulin is associated with lower positive well-being than OADs (P<0.01). Improved insulin administration techniques are associated with higher positive well-being than using a syringe for insulin administration (P<0.01) [19,25]. Strengthening the survival skills of a person living with diabetes assists in managing diabetes and insulin distress, resulting in improved emotional and physical well-being [10,26].

Various sociodemographic characteristics have been associated with lower HRQOL in both type 1 and type 2 diabetes, according to the studies [13,27]. According to studies, older patients experience less diabetes distress than younger patients, because they are more positive, have a lower self-perception of disease-related suffering, and adhere to self-management programs such as diet, exercise, and frequent blood glucose monitoring [7,28-29]. Moreover, the influence of various factors diminishes with age, and some HRQOL domains are concentrated in older age ranges in both people with or without diabetes [27,30-31]. In our study, a greater number of patients in the higher age group reported GH and the results were found to be statistically significant. There are contradictory findings regarding the link between HbA1c levels and well-being scores, with some studies finding no relationship [32-33] and others finding one. A study by Alvani et al., which included 1000 patients of T2D found that the W-BQ 22 scale did not show any correlation between HbA1c levels and well-being scores [34]. In another study by Berry et al., it was found that diabetes distress was significantly linked to higher HbA1c levels [13]. In addition, high PAID and DDS11 scores for diabetic distress were found to have a significant correlation with higher HbA1c levels [13,35]. In our study, we found a significant positive association between the reduction in HbA1c, FPG, and PPG levels as well achievement of GH. In addition, our study revealed a significant negative association between GH and smoking and drinking behavior. Previous studies have highlighted the importance of accepting substance use behavior as part of the overall health status and well-being of diabetes patients [36]. Furthermore, studies have found that drinking alcohol is linked to poor adherence to T2D care regimens [36].

Strength and limitations

The key limitation of the study is the inadequate representation of both urban and rural populations to accurately portray the impact of geographical and socioeconomic status on GH. The missing data in current study may have potential to introduce the bias. The presence of other potential confounders such as education level, financial status and medication adherence could influence GH scores. Hence, these confounders should be adjusted in future study to enhance the reliability of GH scale. Additionally, the GH scale used in this study had been adapted from validated scores rather than being independently validated before this study. Consequently, the results are only indicative of the trends of satisfaction or dissatisfaction. Furthermore, the lack of data for HbA1c, FPG, and PPG in a few patients has hampered the assessment of GH's connection with glycemic control. In addition, a few other characteristics related to GH, such as social behavior, physical activity, mindfulness training, and nutrition, were not evaluated in this study. The proposed future direction is to conduct a study with a larger sample size to validate the GH scale by analyzing the internal consistency and test-retest reliability comparisons with existing tools. Additionally, conducting the longitudinal study in future may confirm the causality between GH score and patient associated factors such as glucose levels and dosing frequency of OAD.

## Conclusions

Various factors such as high glucose levels and OAD dosage frequency have been negatively associated with GH in patients with T2D. As a result, to attain long-term contentment and well-being in managing diabetes, there is a need to focus on various aspects of GH among both patients and their physicians.

## Appendices

**Table 7:** GH scale - Patient Component 40-50: Happy; 30 to < 40: Neutral; 0 to < 30: Unhappy

Sr. No.	Sentiment level	1	2	3	4	5
Questionnaire		Strongly Disagree	Disagree	Neutral	Agree	Strongly Agree
1	Are you satisfied with your understanding of your diabetes?					
2	Do you feel that friends or family appreciate the difficulty in living with diabetes?					
3	Do you feel that you are happy and satisfied with your life presently?					
4	How flexible have you been finding your treatment to be recently?					
5	How convenient have you been finding your treatment to be recently?					
6	How confident do you feel to manage your blood sugar level when it goes higher or lower than it should be?					
7	Do you feel your private and social leisure activities are not affected due to diabetes?					
8	Do you feel that diabetes is not affecting your mental and physical energy every day?					
9	Do you feel that you can cope up with depression or fear when you think about living with diabetes?					
10	Do you feel that you are unaffected by the demands of living with diabetes?					
	Total Score					

**Table 8:** GH scale - Physician Component 40-50: Happy; 30 to < 40: Neutral; 0 to < 30: Unhappy

Sr. No.	Sentiment level	1	2	3	4	5
Questionnaire		Strongly Disagree	Disagree	Neutral	Agree	Strongly Agree
1	Do you feel happy and satisfied that you chose to be a diabetes care professional?					
2	Do you get satisfaction from being able to help T2DM patients?					
3	Do you feel you can make a difference in life ofT2DM patients through your work?					
4	Do you feel that you are physically and emotionally replenished at work?					
5	Do you feel that you are excited to manage the T2DM patients at work?					
6	Do you feel you are in control dealing with complex problems of T2DM management?					
7	Do you feel that you are effectively performing your job as a Health care provider for T2DM patients?					
8	Do you feel that you are able to manage the higher load of T2DM patients?					
9	Do you feel that you are mentally prepared to deal the traumatic stress of T2DM patients whom you try to help?					
10	Do you feel empathetic and connected with your colleagues and friends?					
	Total Score					
